# Advances in Polyhydroxyalkanoate (PHA) Production, Volume 2

**DOI:** 10.3390/bioengineering7010024

**Published:** 2020-03-04

**Authors:** Martin Koller

**Affiliations:** 1 Institute of Chemistry, University of Graz, NAWI Graz, Heinrichstrasse 28/VI, 8010 Graz, Austria; martin.koller@uni-graz.at; Tel.: +43-316-380-5463; 2 ARENA—Association for Resource Efficient and Sustainable Technologies, Inffeldgasse 21b, 8010 Graz, Austria

**Keywords:** biomedical application, cyanobacteria, feedstocks, gaseous substrates, haloarchaea, high cell density cultivation, in-line monitoring, PHA composition, PHA processing, polyhydroxyalkanoate, process engineering, process simulation, *Pseudomonas* sp., rheology, terpolyester, waste streams

## Abstract

During the two years that have passed since the first volume of “Advances in Polyhydroxyalkanoate (PHA) production” was published, the progress in PHA-related research was indeed tremendous, calling for the next, highly bioprocess- and bioengineering-oriented volume. This editorial paper summarizes and puts into context the contributions to this second volume of the *Bioengineering* Special Issue; it covers highly topical fields of PHA-related R&D activities, covering, beside the pronounced bioengineering-related articles, the fields of the microbiology of underexplored, but probably emerging, PHA production strains from the groups of *Pseudomonas*, cyanobacteria, methanotrophs, and from the extremophilic domain of haloarchaea. Moreover, novel second-generation lignocellulose feedstocks for PHA production from agriculture to be used in biorefinery concepts, new approaches for fine-tuning the composition of PHA co- and terpolyesters, process simulation for PHA production from methane-rich natural gas, the challenges associated with rheology-governed oxygen transfer in high cell density cultivations, rapid spectroscopic in-line analytics for process monitoring, and the biomedical application of PHA biopolyesters after appropriate advanced processing are the subjects of the presented studies.

## 1. Introduction

Nowadays, we are witnessing highly dynamic research activities in the captivating field of biodegradable materials with plastic-like properties. These activities are indeed boosted by an increasing public awareness of prevailing ecological issues connected to growing piles of plastic waste, the microplastic predicament, new regulations for plastic use and management, and increasing greenhouse gas emissions contributing to global warming and climate change. These environmental concerns go in parallel with the continuing depletion of fossil feedstocks, which are used at present to produce established full carbon backbone plastics. To a gradually increasing extend, polyhydroxyalkanoate (PHA) biopolyesters, a family of microbial storage compounds with versatile plastic-like material properties, are considered a future-oriented answer to these problems. Bio-catalyzed PHA production is based on renewable resources, and occurs in vivo by the action of living prokaryotic cells. If performed in an optimized way, both PHA production and the entire lifecycle of PHA-based products are embedded into nature´s closed carbon cycle [1]. Still, experts in this field emphasize that sustainable and efficient PHA production requires the understanding and optimization of all individual process steps [2]. The holistic improvement of PHA production, applicable also on an industrial scale, *inter alia* calls for: optimized bioprocess engineering and adapted fermentation modes [3], consolidated knowledge about the enzymatic, metabolic, and genetic ongoings in PHA accumulating organisms in the context of “Next Generation Industrial Biotechnology” [4], the multi-facetted role of PHA granules in living cells and the impact of environmental stress factors on PHA formation [5], an in-depth understanding of the kinetics of the bioprocess [6], the selection of ethically clear, inexpensive feedstocks [7,8], tailoring the composition of PHA on the level of the monomeric constituents [9], and efficient and ecologically benign strategies for PHA recovery from biomass [10].

Since the publication of the first *Bioengineering* Special Issue, “Advances in Polyhydroxyalkanoate (PHA) production”, two years have passed [11]. During this period, global R&D activities in the field of PHA biopolyesters have increased at a breathtaking pace, with several new ambitious and highly specialized research groups now having succeeded to become global trendsetters in this scientific field. In view of the apparently exponentially growing number of relevant publications, it is actually a challenging task to completely and permanently keep up with all the ongoing developments in PHA research. Hence, it is definitely time for an update on the *status quo* of PHA research! Therefore, this Special Issue of articles addresses several distinguished aspects of the PHA production chain; it consists of a total of eleven original articles and specialized review papers written by globally recognized experts who have had a significant impact on this research area already for a long time, and by emerging groups who have only recently consolidated their scientific position in the PHA community.

## 2. Individual Contributions

The search for novel, inexpensive, and amply available feedstocks for ethically clear and economic PHA production is a research direction that is still heavily pursued. In this context, the Mexican research group of Yolanda González-García and colleagues resorted to tequila agave bagasse (TAB), which constitutes the lignocellulosic, fibrous waste stream originating from tequila production. TAB accrues in large amounts and currently has to be disposed of somehow. Therefore, the use of hydrolyzed TAB as a source of hexose and pentose sugars for the production of poly(3-hydroxybutyrate) (PHB) homopolyester by the strain *Burkholderia sacchari* was studied. For this purpose, TAB was chemically hydrolyzed to xylose (pentose) and glucose (hexose) as the main sugars. Next, the effect of hydrolysis by-products, such as phenolic compounds, on *B. sacchari* growth was evaluated. After the removal of those inhibiting phenolics by convenient methods, the detoxified hydrolysate was used as feedstock for PHB production in a two-step batch cultivation process, resulting in a PHB concentration of almost 3 g/L after 122 h of cultivation. This study provides another example how PHA production can be integrated into the production lines of existing agricultural and food-processing industries in order to upgrade existing waste streams to feedstocks for biopolymer production [12].

Dan Kucera and co-workers optimized the production of PHA terpolymers with enhanced material properties consisting of 3-hydroxybutyrate (3HB), 3-hydroxyvalerate (3HV), and 4-hydroxybutyrate (4HB) using the short-chain length (*scl-*) PHA producer *Cupriavidus* sp. DSM 19379. Cultivations in presence of the 4HB-precursor compounds γ-butyrolactone (GBL), ε-caprolactone, 1,4-butanediol, and 1,6-hexanediol resulted in the biosynthesis of PHA copolyesters consisting of 3HB and 4HB building blocks. Furthermore, single- and two-stage production strategies were tested for the production of terpolyesters consisting of 3HB, 3HV, and 4HB monomers. During single-stage cultivation, GBL and 1,4-butanediol served as the main substrates, while propionic and valeric acid acted as 3HV-precursors. During two-stage production, glycerol was used in the phase of biomass growth, while 3HV- and 4HB-precursors for terpolyester formation were used in the second, nitrogen-limited, cultivation phase. The obtained terpolyesters contained 0%–29% mol 3HV and 16%–32% mol 4HB and were thoroughly characterized by state-of-the art analytical tools in order to assess their thermo-mechanical properties and molecular mass distribution in dependence on the PHA composition [13].

Warren Blunt and associate researchers addressed the important topic of high-cell-density cultivations, which are a pre-condition for high-throughput, high-productivity PHA production. However, high-cell-density cultivations pose bioengineering challenges; in the bioreactor, the increasing viscosity severely impacts oxygen import by making mixing and sparging more difficult. To get detailed knowledge about the impact of high-cell-density fed-batch cultivation on the kinetics of PHA-accumulating strain *Pseudomonas putida* LS46, the time-dependent rheological properties of the microbial cultures were followed during cultivations. It was shown that increasing cell density drastically increases culture viscosity, making the cultivation broth increasingly shear-thinning. However, at increasing shearing rates, shear-thickening behavior was observed. It was shown that the cell-free supernatant contributed more to observed viscosity than the cells themselves. As a consequence, the oxygen transfer performance of the bioreactor system dropped to only 50% of the performance at the beginning of the cultivation, showing that the dynamic rheological behavior of high-cell-density cultures is a pivotal process engineering parameter to be considered, which severely impacts the success of PHA production processes in terms of the productivity, scalability, and efficiency of downstream processing [14].

High-cell-density fed-batch cultures of *Pseudomonas putida* LS46 were also studied in the second article by W. Blunt et al., who tested a reactive pulse feeding approach based on real-time measurements of CO_2_ and dissolved oxygen (DO) as feedback variables. This way, an oxygen-limited fed-batch process for enhanced medium chain length (*mcl*-) PHA productivity was developed. Using octanoic acid as the sole carbon source in a bioreactor operated under atmospheric conditions, about 30 g/L cell dry mass (CDM), containing about 60 wt.% of *mcl*-PHA (volumetric productivity: about 0.7 g/(L·h)) was obtained within 27 h of operation, although the onset of oxygen limitation occurred already 14 h after start of the process. While supplying the carbon source via a “continuous drip feed process” (determined as a “proactive feeding strategy” in contrast to pulse feeding) did not significantly impact the final volumetric productivity, it favored the production of non-PHA biomass during bacterial growth, while pulse feeding boosted *mcl*-PHA concentration and product yield during the accumulation phase. Hence, intrinsic O_2_-limitation in high-cell-density cultivation setups can be implemented as a convenient and efficient control tool for enhanced *mcl*-PHA synthesis from long chain carboxylic acids. Furthermore, the pulse feed strategy turned out as a reliable and relatively easy strategy for quick optimization of fed-batch processes, especially in the case of rather toxic substrates like octanoic acid [15].

In the context of *mcl*-PHA, Grazia Licciardello and associated scientists comprehensively reviewed the co-production of elastomeric *mcl*-PHA and extracellular products (EPS, mainly alginates) both on related and unrelated carbon sources by strains of *Pseudomonas corrugata* and *P. mediterranea*. It is shown that product yield and product composition are dependent on the production strain, the carbon source, the cultivation process, and fermentation additives. Selected *P. corrugata* strains accumulate amorphous and sticky *mcl*-PHA from high-grade and partially refined glycerol, a by-product of the biodiesel production process. In contrast, *P. mediterranea* strains produce a characteristic filmable PHA, very different from typical microbial *mcl*-PHA. These novel *mcl*-PHA films are suitable for manufacturing biopolymer blends with polylactic acid (PLA). As a drawback, *mcl*-PHA yields still need to be increased, and production costs to be reduced. Furthermore, an integrated process is presented in this review to conveniently recover intracellular *mcl*-PHA by the halogen-free solvent acetone and bioactive EPS by chloroform/methanol mixtures. Moreover, available transcriptional regulation studies during PHA production contribute to better understand the metabolic potential of *P. corrugata* and *P. mediterranea* strains; here, the available data suggest that regulating of PHA biosynthesis genes will enable to develop new, integrated strategies for cost-effective *mcl*-PHA and EPS production in the future [16].

Björn Gutschelmann and colleagues addressed the fact that effective process development and monitoring is needed for competitive PHA production; this can be achieved by appropriate on-line or in-line monitoring devices. In this context, photon density wave (PDW) spectroscopy was tested for the first time as a new process analytical tool (PAT) for the cultivation of *Cupriavidus necator* H16 on plant oils for PHA production. PDW spectroscopy was used for the in-line monitoring of the reduced scattering coefficient µ_s_’ and the absorption coefficient µ_a_ at 638 nm. A correlation of µ_s_’ with CDM and µ_a_ with the non-PHA biomass was observed during the phases of growth, PHA accumulation, and PHA degradation in batch and fed-batch cultivation setups. These correlations were successfully used to forecast CDM and the PHA content in a high cell density fed-batch cultivation process with a volumetric PHA productivity of 1.65 g/(L∙h), a CDM of 106 g/L, and a PHA content in biomass of 73 wt.% [17].

Mohsen Moradi and associate researchers simulated the ability of special microorganisms for PHB production using methane-rich natural gas as a carbon source in a bubble column bioreactor. Using the Taguchi algorithm, the optimum dimensions (length and diameter) of the bubble column and process conditions for PHB production from natural gas were evaluated as 30 cm length, 1.5 cm diameter, and a temperature of 32 °C. Moreover, an optimum volume ratio of air-to-methane of 1:1 was calculated. The simulation was carried out by using the COMSOL software tool with the two-dimensional symmetric mode. Mass transfer, momentum, density-time, and density-place were studied. The maximum biomass concentration amounted to 1.63 g/L, which shows a difference of only 10% in comparison to elaborated experimental results. Moreover, the impact of the inlet gas rate on product concentration and gas hold up was studied, and the simulated results were compared to experimental data; a high prediction performance of the simulation was demonstrated by a deviation of less than 20% [18].

Donya Kamravamanesh and colleagues reviewed PHA production from another gaseous substrate, namely CO_2_-based, solar-driven PHA production by cyanobacteria. Cyanobacteria can accumulate PHA under photoautotrophic growth conditions using CO_2_ and sunlight. It is demonstrated that the productivity of photoautotrophic PHA production from cyanobacteria is considerably lower than that described for the PHA production process by many chemoheterotrophic microbes. Therefore, a lot of effort has been dedicated during the last years to decrease PHA production cost, mainly by developing optimized microbial production strains and more effective cultivation and downstream processes. It is shown that a reduction in the PHA production cost can only be achieved by considering the process design and by a complete, holistic analysis of the entire PHA production process. With the final aim being the market success of PHA, this review discusses the benefits and the challenges associated with the upstream processing of cyanobacterial PHA production with a focus on process engineering aspects, in order to support and accelerate the realization of this promising PHA production approach on an industrial scale. Most of all, it is suggested that cyanobacterial PHA production plants have to be installed next to CO_2_-emmiters, such as power plants or other factories, and should resort to the use of nutrient-rich wastewater. Moreover, robust cyanobacteria should be the production strains of choice to allow their cultivation under septic conditions in open tanks and ponds. Finally, PHA should not be the only product recovered from cyanobacteria; profiting also from other marketable products, such as pigments or bioactive compounds, cyanobacteria should emerge as whole-cell bio-factories and get embedded into biorefinery concepts [19].

Haloarchaea are another particular group of microbes deserving of specific consideration when it comes to PHA biosynthesis. Among them, we find a gradually growing number of microbes that accumulate significant quantities of PHA. These earliest organisms on earth live in challenging habitats at salinities between 100 and 300 g/L NaCl and could potentially beat established PHA production strains in future due to several benefits: cultivations in highly saline media can be run at reduced sterility by preventing the growth of non-halophilic contaminants; the high intra-cellular osmotic pressure of haloarchaea simplifies the release of intracellular PHA granules by hypo-osmotic cell disintegration; many haloarchaea convert diverse inexpensive carbonaceous waste materials as feedstocks for growth and PHA production, which combines PHA production with waste upcycling; some haloarchaea are even said to produce high-quality copolyesters from simple, structurally unrelated inexpensive substrates; finally, PHA biosynthesis often takes place in parallel to the biosynthesis of additional marketable compounds, such as polysaccharides, antibiotics, or pigments. The current knowledge on PHA production by haloarchaea is reviewed in this article, covering the quest for new PHA-producing haloarchaea, their genetic and enzymatic idiosyncrasies, the properties of haloarchaeal PHA, successful attempts for upscaling PHA production by haloarchaea, and techno-economic and life cycle assessments of selected processes [20].

In the context of emerging biomedical applications of PHA, Alejandra Rodríguez-Contreras reviewed that their central characteristics biocompatibility, biodegradability, and non-toxicity are the pivotal properties that make them suitable for applications as biomaterials. It is discussed how PHA has been used as bone graft substitutes, in tissue engineering, as sutures, as valves in implantology, for cartilage repair, to develop new biological stents for nerve repair, and for cardiovascular patches. Based on their expedient in vivo biodegradability and the fact that the products of their in vivo degradation are non-toxic, PHA have also been broadly used as biological carriers for fine-tuned drug-release systems. Due to the fact that global interest in the biomedical application of PHA is steadily increasing, this review shines a light on the most recent scientific outcomes and advances in the exploitation of PHA and their follow-up products as biomaterials in diverse medical fields [21].

Finally, Dario Puppi and colleagues addressed the fact that this quickly growing interest on PHA processing for biomedical applications is based on the unique combinations of their characteristics in terms of biocompatibility, biodegradability, processing properties, and mechanical behavior; these indisputable benefits, however, need to go in parallel with their sustainable production. The comprehensive review article presents the best exploited processing techniques up to date employed in the biomedical area to develop devices and other biomedical PHA-based items, both for experimental and commercial applications. To this end, PHA’s physical, thermomechanical, and processing properties are linked to the requirements of processing techniques conventionally employed to process plastics, such as solvent casting or melt-spinning, and to advanced, currently emerging manufacturing techniques, such as electrospinning or additive manufacturing (3D-printing). Key publications on different aspects affecting the workability of differently composed PHA homo- and copolyesters are summarized [22].

## 3. Conclusions

The respected reader will get an insight into current research activities dedicated to the individual scientific fields involved in making PHA biopolyesters market-fit, starting from the exploration of novel microbial production strains, switching to the challenges associated with bioprocess engineering for PHA production, and ending with advanced manufacturing techniques to produce high-performance biomaterials. I am confident that reading this second PHA-related *Bioengineering* Special Issue at hand will lead to a real motivation and inspiration boost for researchers all over the planet; expected future research activities will further deepen our understanding of PHA metabolism, biosynthesis, and its functional role. Most of all, additional efforts will hopefully be dedicated to the improvement of the PHA-related bioengineering as the *conditio sine qua non* for making PHA ultimately competitive on the plastic market.
